# The Effect of Copper and Selenium Nanocarboxylates on Biomass Accumulation and Photosynthetic Energy Transduction Efficiency of the Green Algae *Chlorella Vulgaris*

**DOI:** 10.1186/s11671-017-1914-2

**Published:** 2017-02-23

**Authors:** Natalia F. Mykhaylenko, Elena K. Zolotareva

**Affiliations:** 0000 0004 0385 8977 grid.418751.e M.G. Kholodny Institute of Botany of the National Academy of Sciences of Ukraine, 2 Tereshchenkivska str, Kyiv, 01601 Ukraine

**Keywords:** Copper, Selenium, Nanoparticles, Nanocarboxylates, *Chlorella vulgaris*, Green algae, Productivity, Chlorophyll fluorescence

## Abstract

Nanoaquachelates, the nanoparticles with the molecules of water and/or carboxylic acids as ligands, are used in many fields of biotechnology. Ultra-pure nanocarboxylates of microelements are the materials of spatial perspective. In the present work, the effects of copper and selenium nanoaquachelates carboxylated with citric acid on biomass accumulation of the green algae *Chlorella vulgaris* were examined. Besides, the efficiency of the reactions of the light stage of photosynthesis was estimated by measuring chlorophyll *a* fluorescence. The addition of 0.67–4 mg L^−1^ of Cu nanocarboxylates resulted in the increase in *Chlorella* biomass by ca. 20%; however, their concentrations ranging from 20 to 40 mg L^−1^ strongly inhibited algal growth after the 12th day of cultivation. Se nanocarboxylates at 0.4–4 mg L^−1^ concentrations also stimulated the growth of *C. vulgaris*, and the increase in biomass came up to 40–45%. The addition of Se nanocarboxylates at smaller concentrations (0.07 or 0.2 mg L^−1^) at first caused the retardation of culture growth, but that effect disappeared after 18–24 days of cultivation. The addition of 2–4 mg L^−1^ of Cu nanocarboxylates or 0.4–4 mg L^−1^ of Se nanocarboxylates caused the evident initial increase in such chlorophyll *a* fluorescence parameters as maximal quantum yield of photosystem II photochemistry (*F*_v_/*F*_m_) and the quantum yield of photosystem II photochemistry in the light-adapted state (*F*_v_'/*F*_m_'). Photochemical fluorescence quenching coefficients declined after 24 days of growth with Cu nanocarboxylates, but they increased after 6 days of the addition of 2 or 4 mg L^−1^ Se nanocarboxylates. Those alterations affected the overall quantum yield of the photosynthetic electron transport in photosystem II.

## Background

Nowadays, the range of nanomaterial utilization in biological applications broadens rapidly. Uptake, translocation and accumulation of nanoparticles in living organisms depend on the concentration, kind, size, surface area, chemical composition and stability of nanoparticles, species of organism etc. [1–3]. Interaction of nanoparticles with organisms may cause various physiological and biochemical changes, both positive and negative.

Promising nanomaterials are not only colloid solutions of nanoparticles but also so-called nanoaquachelates, the nanoparticles with the molecules of water and/or carboxylic acids as ligands. Primary nanomaterials for nanoaquachelate production are colloid nanoparticles obtained by means of blast erosion method, the type of electric impulse ablation technique based on the effect of energy self-concentration in the local microvolumes of conductor [4, 5]. Blast erosion nanotechnologies provide the opportunity to produce chelates with high coordination numbers [4]. The stability of such chelate complexes does not depend on the dimensions of nanoparticles because the surface electric charge and, correspondingly, the coordination number of the obtained spherical nanoparticle are proportional to its size. The obtained hydrated nanoparticles possess high chemical activity and hence can be further carboxylated by addition of the appropriate carbonic acid [6]. The structure of nanoaquachelates can be described by general formula [*ηM* ^2*n*−^(H_2_O)_*m*_(HOOCR)_*p*_]^2*n*−^, where *ηM* ^2*n−*^ is a core nanoparticle with surface electric charge *2n−*; *m* and *p* are the numbers of H_2_O and RCOOH ligands, respectively [7]. The value of the negative electric charge at the surface of nanoparticle relates to the amount of ligands as 2*n =* 2*m + p*. The ability of hydrated and/or carboxylated nanoparticles to penetrate easily the cell membranes and release the ligands thereafter form the prerequisite for their high biological activity coupled with biocompatibility. Another characteristic feature of resultant products is their extremely low content of impurities, as compared with the nanomaterials obtained by chemical methods [4]. It was found that the toxicity of metal nanoaquachelates was much lesser than that of respective inorganic salts [4, 7]. Currently, the ultra-pure nanocarboxylates of principal biogenic and biocide elements are produced commercially and used widely in human and veterinary medicine, agriculture, food and cosmetic industry, municipal engineering, etc. [4]. Good prospects have nanocitrates, so far as the salts of citric acid are approved for use in the food industry [8].

Copper (Cu) and selenium (Se) are microelements essential for plant metabolism; however, their higher concentrations exert toxic effects. The ranges of physiological Cu and Se concentrations are rather narrow; they depend on the type of microelement-containing compound, oxidation state of microelement, species of the organism and many other factors. Copper is involved in many physiological processes because it can exist in multiple (Cu^2+^ and Cu^+^) oxidation states in vivo. Copper acts as a structural element in regulatory proteins and participates in photosynthetic electron transport, mitochondrial respiration, oxidative stress responses, cell wall metabolism and hormone signaling [9]. Copper ions act as cofactors in many enzymes such as Cu/Zn superoxide dismutase, cytochrome *c* oxidase, plastocyanin, amino oxidase, laccase and polyphenol oxidase [9]. At the cellular level, copper also plays an essential role in signaling of transcription and protein trafficking machinery, oxidative phosphorylation and iron mobilization [9].

Selenium is mostly involved in antioxidative processes [10]. This trace element is indispensable not only for heterotrophic organisms, such as mammals, fish and many bacteria, but also for certain green algae. However, its physiological significance for other photosynthesizing organisms, including higher plants, is not ascertained finally [11]. Predominant forms of bioavailable Se are its inorganic salts, selenite (SeO_3_^2−^) and selenate (SeO_4_^2−^). Living organisms contain selenium mostly in the form of selenoproteins, where Se atoms replace those of sulphur in some cysteine and methionine residues. In particular, Se is found in the active centres of such antioxidant enzymes as glutathione peroxidases and thioredoxin reductases [12]. At the same time, high concentrations of selenium (1–10 mg kg^−1^ for many plant species) are toxic, due to excessive incorporation of Se in place of S in amino acids, with subsequent alteration of protein three-dimensional structure and impairment of their enzymatic functions [11, 13].

The aim of the present research was to study the effects of copper and selenium nanoaquachelates carboxylated with citric acid on the growth rates and efficiency of photosynthetic photochemical reactions of the unicellular green algae *Chlorella vulgaris* that are widely used in biotechnology.

## Methods

*C. vulgaris* Beijer. was grown under sterile conditions at a temperature of 25–26 °C in 1000-mL Erlenmeyer flasks containing 400 mL of liquid mineral medium [14], EDTA being omitted:

**Table:** 

	[mg L^−1^]
KNO_3_	5000
MgSO_4_·7H_2_O	2500
KH_2_PO_4_	1250
FeSO_4_·7H_2_O	9
H_3_BO_3_	2.86
MnCl_2_·4H_2_O	1.81
ZnSO_4_·7H_2_O	0.222
MoO_3_	0.018
NH_4_VO_3_	0.023

The cultures were illuminated continuously with warm white fluorescent lamps providing the irradiance of 40–42 μmol m^−2^ s^−1^ photosynthetic photon flux density and stirred by shaking two times a day. At inoculation, the algal cultures were supplied with copper or selenium nanoparticles (average size about 100 nm) carboxylated with citric acid (nCu-Citr or nSe-Citr, respectively) [6, 7], obtained from the Ukrainian State Scientific Research Institute ‘Resource’ (Kyiv, Ukraine), to final concentrations ranging from 0.67 to 40 mg L^−1^ (nCu-Citr) and from 0.07 to 4 mg L^−1^ (nSe-Citr). Algal culture without nanoparticle addition was used as a control one. The samples for analyses were harvested at the beginning of the experiment and then every 6 days.

To determine the dry mass of algae, they were concentrated by centrifugation (10 min at 1500×*g*), washed two times with distilled water with the subsequent biomass concentration and dried to a constant weight at 105 °C.

The efficiency of photochemical reactions in the photosystem II was estimated by measuring modulated chlorophyll *a* fluorescence at room temperature [15] using the fluorometer XE-PAM (Heinz Walz GmbH, Effeltrich, Germany). Prior to measurements, all the samples were dark-adapted for 10 min in the stirring-enabled fluorometer cuvette. The measuring light flashes (2 Hz, 0.15 μmol m^−2^ s^−1^) were adjusted to be sufficiently weak for the prevention of photochemical charge separation in photosystem II reaction centres, as described previously [16]. The actinic light intensity was similar to that applied during the cultivation of algae. The saturating pulse duration and intensity (5500 μmol m^−2^ s^−1^, 1 s) were selected to ensure the complete closure of all photosystem II reaction centres.

Common chlorophyll fluorescence parameters were calculated [15, 17]:

- *F*_v_/*F*_m_, maximal quantum yield of photosystem II photochemistry
- *F*_v_'/*F*_m_', quantum yield of photosystem II photochemistry in the light-adapted state
- *q*_P_, or *F*_q_'/*F*_v_', photochemical quenching coefficient
- *q*_L_, or (*F*_q_'/*F*_v_')(*F*_0_'/*F*'), photochemical quenching parameter estimating the fraction of open photosystem II centres
- NPQ, non-photochemical quenching
- *Φ*_PSII_, or *F*_q_'/*F*_m_', the effective quantum yield of photosystem II electron transport

All the measures were taken as the average of minimum three biological and analytical replicates from different test samples, and standard deviations were calculated.

## Results and Discussion

**Fig. 1:**
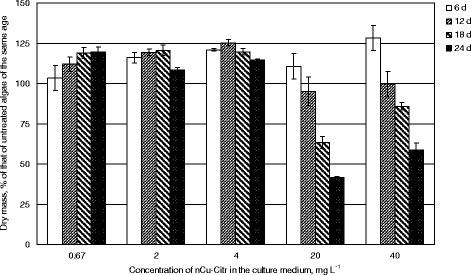
Biomass of *Chlorella vulgaris* grown in the presence of nCu-Citr

The concentrations of copper nanoparticles chosen for the present experiment correspond to those of copper ions naturally occurring in the polluted aquatic environments [18]. The addition of copper nanoparticles carboxylated with citric acid (nCu-Citr) in all the range of concentrations examined evoked the initial increase in *Chlorella* dry matter (Fig. 1). If nCu-Citr concentration did not exceed 4 mg L^−1^, 20% growth stimulation was retained up to the end of experiment. Notably, the effect of the lowest applied concentration (0.67 g L^−1^) was the most sustainable. On the contrary, the addition of 20 to 40 mg L^−1^ nCu-Citr showed to be toxic to *C. vulgaris* because it stopped the algal growth starting from the 12th day of cultivation.

The increase in inorganic copper concentration may cause the impairment of the biochemical and physiological processes in algal cells [19]. The redox transitions that make Cu an essential element also contribute to its inherent toxicity, catalyzing the production of reactive oxygen species with subsequent damage of biomolecules [9]. However, copper is one of the most toxic metals to unicellular algae [20]; *C. vulgaris* was earlier found to be rather insensitive to copper toxicity [18, 21]. On the one hand, it was shown that *C. vulgaris* growth in the presence of 100 mg L^−1^ of copper was not impaired [22] or that it can tolerate up to 0.5–1 mg L^−1^ [23] or even 100 mM [24] copper. However, other researchers found that the exposure to 0.5–100 μM Cu significantly decreased growth, chlorophyll and protein content, increased reactive oxygen species content and reduced the transcript abundance of photosynthesis-related genes *psb*A and *rbc*L [20, 25, 26].

CuO nanoparticles have been shown to induce growth inhibition and lead to cellular oxidative stress in green alga *Chlamydomonas reinhardtii* at concentrations higher than 100 mg L^−1^ [27]. The nanoparticle form of Cu was proved to have a unique advantage over Cu^2+^ in entering the bacterial cells, thus mediating specific effects (e.g. DNA oxidative damage) [2]. Additional testing should be conducted on the effects of various nano-Cu species in other organisms.

**Fig. 2:**
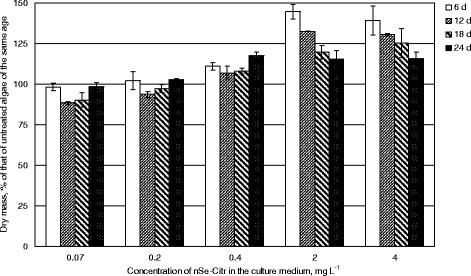
Biomass of *Chlorella vulgaris* grown in the presence of nSe-Citr

Selenium nanoparticles carboxylated with citric acid (nSe-Citr) at 2 or 4 mg L^−1^ concentrations induced 1.4-fold increase in *C. vulgaris* biomass accumulation after 6 days of cultivation (Fig. 2). Their positive effect on *Chlorella* growth retained throughout the whole period of experiment, although weakening with time. If nSe-Citr was added to 0.4 mg L^−1^ concentration, during 18 days, the growth of *C. vulgaris* increased only by 7–11%, but then it accelerated, and at the end of the experiment became the same as in the case of addition of five- to tenfold higher nSe-Citr concentrations. Being added at smaller concentrations (0.07 or 0.2 mg L^−1^), nSe-Citr caused the retardation of culture growth on the 12th day, but the amount of *Chlorella* dry mass gained the values of the control culture on the 18th day (0.2 mg L^−1^ nSe-Citr) or on the 24th day (0.07 mg L^−1^ nSe-Citr).

Inorganic selenium salts in the concentration range close to that applied in the present study can either stimulate or inhibit the growth of various algal species [12, 28–31]. For example, the growth of *C. vulgaris* in Erlenmeyer flasks under the temperature and illumination regime similar to that of our study was stimulated by 25–75 mg L^−1^ sodium selenite [30].

Chlorophyll *a* fluorescence kinetic analysis is commonly used as a tool to identify changes in photosynthetic light reactions because environmental stress can reduce the ability of plants to metabolize normally, resulting in imbalance between the light energy absorption by chlorophyll and the use of energy in photosynthesis [25]. Based on our data concerning *Chlorella* biomass accumulation, only the effects of 0.67–4 mg L^−1^ of nCu-Citr on modulated chlorophyll *a* fluorescence were determined, and for nSe-Citr, the effects of the whole concentration range were examined.

**Fig. 3:**
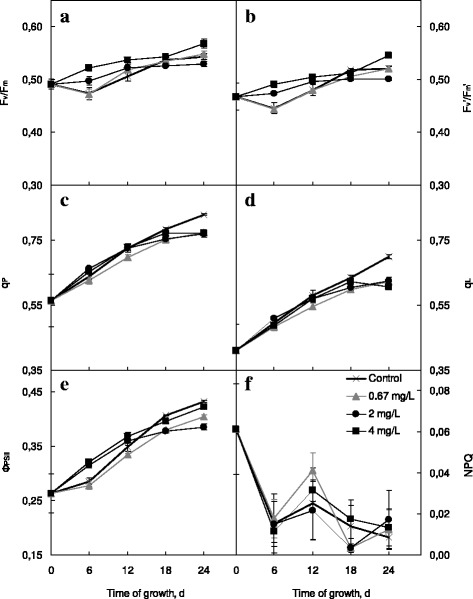
Chlorophyll fluorescence parameters of *Chlorella vulgaris* grown in the presence of various concentrations of nCu-Citr (**a**–**f**)

**Fig. 4:**
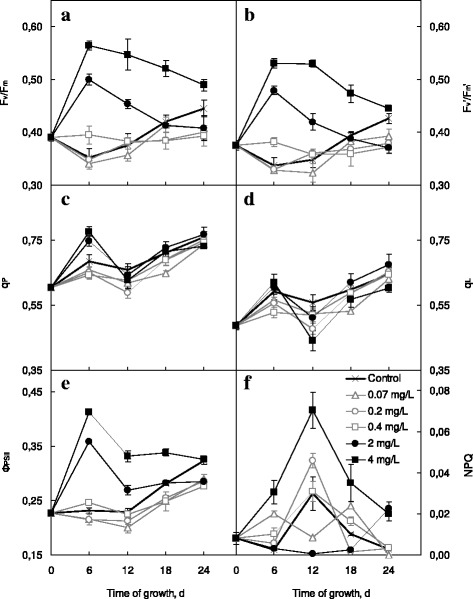
Chlorophyll fluorescence parameters of *Chlorella vulgaris* grown in the presence of various concentrations of nSe-Citr (**a**–**f**)

Copper (2–4 mg L^−1^) and selenium (0.4–4 mg L^−1^) nanocarboxylates caused the initial rise in *F*_v_/*F*_m_ and *F*_v_'/*F*_m_' parameters (Figs. 3 and 4a, b) indicating, respectively, the capacity of dark-adapted and light-adapted algal cells to convert light energy into the energy of chemical bonds. Thereafter, in the case of nCu-Citr addition, the difference of both *F*_v_/*F*_m_ and *F*_v_'/*F*_m_' with the respective control values diminished. On the 24th day of the experiment, their values exceeded the control ones only in the case of 4 mg L^−1^ nCu-Citr added. *F*_v_/*F*_m_ and *F*_v_'/*F*_m_' in *Chlorella* cells grown in the presence of 0.67 mg L^−1^ nCu-Citr showed no appreciable distinctions with those of the control samples. On the other hand, the extent and duration of nSe-Citr positive effect on *F*_v_/*F*_m_ and *F*_v_'/*F*_m_' values increased with the growth of their amount added.

Both photochemical quenching coefficients (*q*_P_ and *q*_L_) estimate the fraction of open photosystem II reaction centres and thus represent the proportion of light excitation energy captured by photosystem II that is used for electron transport. Neither *q*_P_ (Fig. 3c) nor *q*_L_ (Fig. 3d) depended on the concentration of added nCu-Citr, and at the end of experiment, their values were lowered by 7–13% as against the control. On the contrary, 2 or 4 mg L^−1^ nSe-Citr stimulated the increase in *q*_P_ on the sixth day of *C. vulgaris* cultivation (Fig. 4c), and *q*_L_ was lowered after 12 days of *Chlorella* growth with 4 mg L^−1^ nSe-Citr (Fig. 4d). The assessment of *q*_P_ parameter is widely accepted; the formula for *q*_P_ calculation is based on the assumption that each photosystem II centre possesses its own independent antenna system. Otherwise, the *q*_L_ parameter proposed by Kramer et al. [32] proceeds from the so-called lake model that photosystem reaction centres are connected by shared antennae and is recommended for more accurate assessment of the redox state of the primary quinone acceptor (*Q*_A_) pool instead of *q*_P_, especially at high light intensities [17]. In our experiment, even taking into account that photosynthetic photon flux density was rather low, *q*_P_ values exceeded those of *q*_L_ by 15–40% (Figs. 3 and 4c, d). Such results are not common, e.g. in the recent thorough investigation of another green alga, *C. reinhardtii*, undergoing nitrogen deprivation, the values of *q*_L_ were shown to be approximately 1 to 2% lower than *q*_P_ [33]. Taking into account that the calculation of *q*_L_ is easy and does not demand any modifications of the generally accepted fluorescence measurement protocol, more attention may be paid to this parameter in order to gain further insight into the processes of light energy transfer between antennae in algae.

*Φ*_PSII_, or operational photosystem II quantum yield, evaluates the net efficiency of photosystem II photochemical processes and is considered to be the fluorescence index of the rate of linear photosynthetic electron transport. As this parameter is in fact the product of *F*_v_'/*F*_m_' and *q*_P_, therefore, its initial rise in the cells cultivated with 2 or 4 mg L^−1^ nCu-Citr is due to the increase in *F*_v_'/*F*_m_' (Fig. 3e). Similarly, *q*_P_ decline contributes to the lowering of *Φ*_PSII_ on the 24th day of growth in the presence of copper nanoparticles. In the case of nSe-Citr addition, the increase in *Φ*_PSII_ was generally due to the rise of *F*_v_'/*F*_m_' values (Fig. 4e).

The nonphotochemical quenching (NPQ) value represents all quenching processes of the photosystem II chlorophyll fluorescence not directly related to photochemistry [34]. Treatment with copper nanocarboxylates did not affect that parameter significantly in *C. vulgaris* (Fig. 3f). However, the utilization of 4 mg L^−1^ nSe-Citr caused the reliable NPQ increase (Fig. 4f), so one can suggest that absorbed light energy exceeded the capacity of its utilization in photosynthetic processes.

As was found previously [16], millimolar concentrations of citrate do not exert significant effect on photochemical reactions in algae. Therefore, the study of any possible effects of nano- and micromolar citrate concentrations present in *C. vulgaris* cultures was beyond the scope of the present experiment.

Photosynthetic electron transport is known to be altered under both copper deficiency and excess conditions [9, 35, 36]. Photosystem II activity appears to be the most sensitive site of copper inhibition [35, 37, 38]. The important sites of copper inhibitory effect are associated with oxidizing and reducing sides of photosystem II electron transport [18, 37]. Copper ions suppress the activity of photosynthetic water splitting system [37, 39], interact with the Q_B_ [39, 40] and pheophytin [41] electron transport sites, eventually preventing the conversion of light energy absorbed by chlorophyll antenna complex into photosystem II electron transport. The effects of copper on the photosystem II photochemistry consequently decrease carbon dioxide uptake in algal cells [18, 42].

Previously, Juneau et al. [18] found that chlorophyll fluorescence parameters *F*_v_/*F*_m_, *Φ*_PSII_ and *q*_P_ obtained from *C. vulgaris* did not show susceptibility to CuSO_4_ at 100 μg Cu L^−1^ concentrations as compared with other green algae, *C. reinhardtii* and *Selenastrum capricornutum*. Besides, *C. vulgaris* was able to develop resistance to copper if the exposure was prolonged. Similarly, recent investigation by Chen et al*.* [25] showed that 1–2 μM CuCl_2_ somewhat improved photosystem II photochemical performance of *C. vulgaris* (judging by increase in *F*_v_/*F*_m_, *q*_P_ and *Φ*_PSII_ values). At the same time, 4 μM and higher CuCl_2_ provoked plasmolysis of *C. vulgaris* cells accompanied by complete loss of photosynthetic activity, thus emphasizing the extreme narrowness of physiological Cu^2+^ concentration range. In our study, much higher amount of copper in nanocarboxylate form was applied, but the inhibition of photosynthetic energy transduction, if any, was far from prominent.

Little is known to date about the effect of selenium at any form on photosynthetic processes. It was found that selenium (50 mg L^−1^) in the form of sodium selenate stimulated starch accumulation in the cells of the wild type of green alga *Scenedesmus quadricauda*; however, the process of starch hyperaccumulation followed the severe retardation of algal growth [12]. Sodium selenite in the concentrations up to 75 mg L^−1^ raised chlorophyll *a* and carotenoid content after 6 days of *C. vulgaris* cultivation [30]. Selenium was not shown to stimulate photosynthesis of *Euglena gracilis* [29]; however, in that study, selenite was used and its maximal concentration was tenfold lower than in our research. Therefore, the data obtained contribute to the understanding of the mechanisms of selenium effect on the photosynthetic productivity of algae. Further investigations require the thorough study of nanocarboxylate effects on photosynthesis, as compared with inorganic salts of respective microelements.

## Conclusions

The utilization of copper (0.67–4 mg L^−1^) or selenium (0.4–4 mg L^−1^) nanoparticles, carboxylated with citric acid, has positive effect on *C. vulgaris* growth and transiently improves the efficiency of photosystem II photochemical reactions. Therefore, it may be recommended for further investigations aimed to stimulate the accumulation of algal biomass as the source of valuable nutrients, biofuels or soil fertilizers. Since growth and photosynthesis of *Chlorella* tolerate much higher concentrations of microelements in the form of nanocarboxylates than those in ionic forms, nanocarboxylates can be further applied in algal biotechnology.
